# Renal tuberculosis with genitourinary sequelae: a case report

**DOI:** 10.1590/S1678-9946202567022

**Published:** 2025-04-04

**Authors:** Raphael Rocco, Bianca Balzano de la Fuente Villar, Laura da Cunha Ferreira, Remberto Maurício de La Cruz Vargas Vilte, Billy McBenedict, Natalia Chilinque Zambão da Silva, Karla Regina Oliveira de Moura Ronchini, Ianick Souto Martins, Danyelle Cristina de Souza, Patricia Yvonne Maciel Pinheiro, Ezequias Batista Martins

**Affiliations:** 1 Hospital Universitário Antonio Pedro, Serviço de Infectologia, Niterói, Rio de Janeiro, Brazil; 2 Universidade Federal Fluminense, Faculdade de Medicina, Niterói, Rio de Janeiro, Brazil

**Keywords:** Tuberculosis, Mycobacterium tuberculosis, Urogenital, Renal

## Abstract

Urogenital tuberculosis (UGT) constitutes a significant extrapulmonary form of tuberculosis, often presenting non-specific symptoms and a prolonged indolent course that leads to delayed diagnosis and treatment, which can result in severe and irreversible complications such as urinary strictures, renal failure, and infertility. This report describes a case of a 38-year-old man with a five-month history of low back pain, hematuria, dysuria, and altered urinary frequency. Initial treatment for a presumed urinary tract infection failed, and subsequent diagnostic investigations showed stones, nodules, and cysts in his left kidney. A positive tuberculin skin test confirmed the diagnosis of UGT and identification of *Mycobacterium tuberculosis* in urine samples. The patient underwent standard six-month antituberculosis therapy and subsequent retreatment due to persistent symptoms. Despite significant symptom amelioration, irreversible urological sequelae, including infundibular stenosis, polyuria, and nocturia, remained. This case underscores the importance of early suspicion, accurate diagnosis, and timely treatment of UGT to minimize long-term complications. It also highlights the potential need for extended treatment length in complex cases to improve outcomes and reduce sequelae, warranting further research in this area.

## INTRODUCTION

Extrapulmonary tuberculosis accounts for 5 to 45% of tuberculosis (TB) cases globally, from 30 to 40% of which involving the urogenital tract. Following pulmonary TB, 2 to 20% of individuals may develop genitourinary TB, often after a latency period of five to 40 years^1,2^. Of these, urinary TB occurs more often than genital TB. Delayed diagnosis and treatment can result in irreversible complications, including urethral strictures, ureteric strictures, and renal failure, necessitating lifelong specialized care^3^.

Urogenital tuberculosis (UGT) is reported to affect women twice as often as men, although these findings remain contentious due to limited epidemiological and clinical studies. Figueiredo *et al.* ^4^ reported greater predominance in men. This extrapulmonary manifestation is more frequently diagnosed in patients with end-stage renal disease or those who have undergone kidney transplantation^5,6^. The diverse symptoms associated with this form of tuberculosis make diagnosis particularly challenging, and delays in identifying and treating the condition can lead to severe and irreversible complications, such as urinary dysfunction and infertility^6^. This study describes a complex case of renal TB, offering essential insights for the effective management of this extrapulmonary manifestation.

Informed consent was obtained from the participant involved in this study. The local ethics committee reviewed and approved the study and assigned the approval under CAAE Nº 85594124.9.0000.5243.

## CASE REPORT

A previously healthy 38-year-old man from Sao Goncalo city, Rio de Janeiro State, Brazil, had a history of low back pain, hematuria, dysuria, and altered urinary frequency beginning in March 2022. Notably, he showed no typical signs or symptoms of TB, such as fever, night sweats, or weight loss. The patient also reported no history of smoking, alcohol consumption, or other comorbidities.

A urologist evaluated the patient, initially diagnosing him with a urinary tract infection and prescribing him cefuroxime (500 mg twice daily for five days). When the symptoms failed to improve, the urologist recommended levofloxacin (750 mg daily for three days), which slightly alleviated dysuria and low back pain. However, the remaining symptoms persisted, and no further diagnostic tests, such as imaging studies or urine cultures, were conducted during this period.

**Figure 1:**
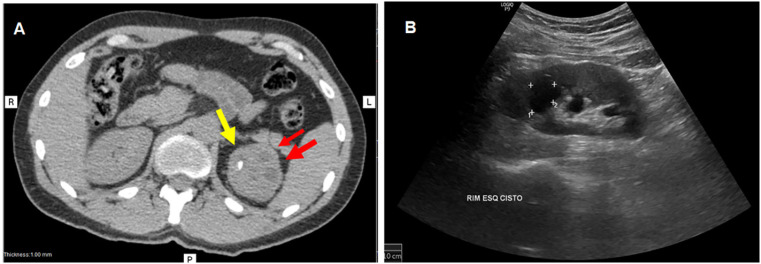
(A) Abdominal CT showing hypodense nodules in the left kidney, a larger one measuring 2.8 × 2.1 cm in the upper third (red arrows) and a smaller nodule measuring 0.7 × 0.3 cm (yellow arrow). A small ectasia of the pyelocaliceal system in this kidney is also observed; (B) Urinary ultrasound showing a cyst in the upper third of the left kidney with parietal calcification and a mixed solid-cystic nodule in the same region.

On May 19, 2022, five months after symptoms onset, the patient was evaluated at a university outpatient clinic with a suspected diagnosis of UGT. He was admitted to a university hospital the following day for diagnostic investigation. Urine cultures, as well as abdominal, pelvic, and lumbar spine computed tomography (CT), were performed, showing nephrolithiasis, solid nodules, and cysts in the left kidney (Figure 1). A renal and urinary tract ultrasound conducted on May 24, 2022, found a calcified nodule in the left kidney along with mild ectasia of the pelvic-calyceal system. A chest X-ray showed no abnormalities. Urine testing performed on admission showed microhematuria (20 red blood cells per high power field) without pyuria.

On May 25, 2022, a tuberculin skin test yielded a positive result, with an induration of 15 mm. On the same day, sputum smear microscopy, urine samples for mycobacterial culture, and a rapid molecular test for tuberculosis were requested to further investigate the suspected diagnosis.

On May 25, 2022, due to strong suspicion of UGT, empirical treatment was initiated with rifampicin (750 mg/day), isoniazid (375 mg/day), pyrazinamide (2,000 mg/day), and ethambutol (1,375 mg/day), planned for a duration of six months (the first two months with the four drugs, followed by four months with only rifampicin and isoniazid). After one week of treatment, the patient experienced significant improvement in his initial symptoms, with no further complaints of hematuria, dysuria, or low back pain. Consequently, he was discharged from the hospital on May 30, 2022, for outpatient follow-up.

On July 12, 2022, during a outpatient follow-up consultation, diagnostic tests performed during hospitalization confirmed the diagnosis of UGT. Ziehl-Neelsen staining of a urine sample identified five acid-fast bacilli, and culture results confirmed the presence of *Mycobacterium tuberculosis*, detecting no rifampicin resistance. However, the rapid molecular test was negative. Hematological and biochemical tests, including kidney and liver function evaluations, were normal during both hospitalization and follow-up. Although the six-month treatment was completed, the patient continued to report changes in urinary frequency and mild pelvic pain.

**Figure 2:**
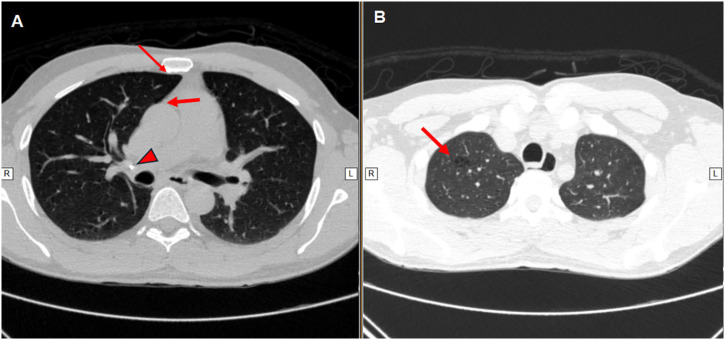
(A) Chest CT showing a calcified nodule in the right hilum (arrowhead), paraseptal emphysema (arrows), and bronchiectasis in the anterior segment of the right upper lobe; (B) Centrilobular emphysema in the apical segment of the right upper lobe and esophageal dilation.

On May 16, 2023, six months after completing treatment, the patient presented with involuntary weight loss (5 kg over one month), dry cough, asthenia, and dysuria, but no fever or sputum production. On the same day, he was readmitted to the same university hospital to assess for recurrent UGT and to rule out other potential conditions, such as neoplasms. Chest CT (May 17, 2023) showed paraseptal and centrilobular emphysema in the upper lobes of both lungs (Figure 2). Abdominal CT performed the same day showed a normal-sized left kidney with nodules in the medullary region, cortical thinning, and no signs of hydronephrosis. A urinary ultrasound (May 18, 2023) showed multisegmental dilation of the calyces in the left kidney indicating chronic stenosis. Since the radiological findings indicated sequelae, the urologist recommended retreatment for UGTB.

Although laboratory tests showed no evidence of TB recurrence (negative urine tests), retreatment for UGT began on May 25, 2023, following the same previously prescribed regimen for a planned duration of six months. By September 2023, after four months of treatment, the patient reported amelioration of most symptoms, with only polyuria and nocturia persisting.

**Figure 3:**
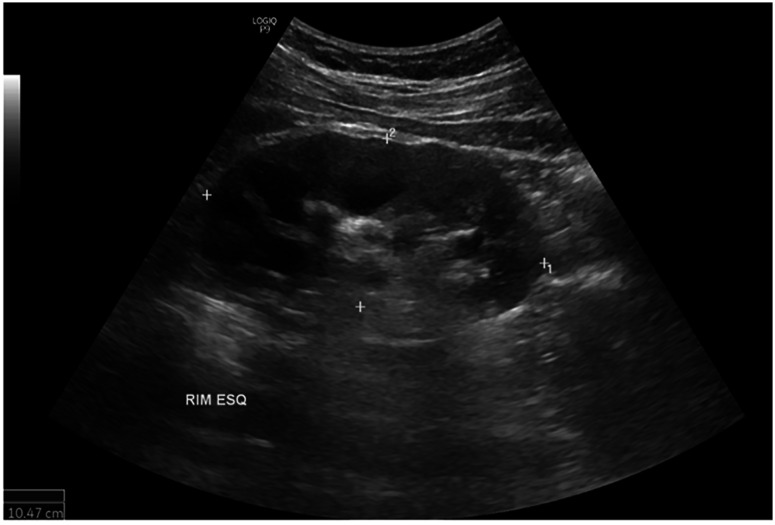
Urinary ultrasound showing multisegmental dilation of the renal calyces on the left, most prominent in the upper third, causing mild cortical thinning in this region. The findings suggest chronic multifocal infundibular stenosis associated with involvement of the collecting system.

A urinary ultrasound performed on September 18, 2023, showed infundibular stenosis in the left kidney, confirming permanent sequelae caused by UGT (Figure 3). Despite completing the six-month retreatment, the patient continued to experience urinary sequelae, including polyuria and nocturia, which significantly impacted his ability to perform his duties as a residential condominium receptionist.

## DISCUSSION

Renal tuberculosis typically arises from the reactivation of a latent renal parenchymal focus, most commonly affecting a single kidney (unilateral). Its latency period varies, the reactivation of which is often linked to immunosuppression. Factors such as alcoholism, malnutrition, AIDS, and diabetes mellitus have been identified as contributing conditions^4^. According to Figueiredo *et al*.^7^, the reactivation of latent renal TB can also be associated with ureteral obstruction due to stones. This obstruction increases intrarenal pressure and ischemia and creates conditions that can activate renal TB foci^7^. In the case above, the presence of stones, nodules, and moderate obstruction in the left kidney supported the development of UGT.

A recent review highlighted that the standard diagnostic methods for UGT include identifying *Mycobacterium* spp. in urine or bodily fluids and biopsy samples cultured on conventional solid media^8^. Imaging modalities, such as X-rays, urinary ultrasound, urography, computed tomography, and magnetic resonance imaging, are invaluable to determine the infection site, guiding tissue sampling, planning medical or surgical treatment, and monitoring therapeutic response^8^. Chest imaging exams are essential for the suspected diagnosis of pulmonary TB and can configure the first step in investigating other forms of the disease^9^. CT has been the most important tool to diagnose and assess sequelae Pulmonary TB^10^. Urogenital findings on CT are the most important tool for suspecting UGT, especially in the absence of pulmonary involvement. In this case, urine sample analysis confirmed the diagnosis, detecting the presence of *M. tuberculosis*. Imaging studies were critical in pinpointing the infection site, finding nodules in the left kidney. The images suggesting sequelae of pulmonary TB in this patient offered a useful element to consider UGT.

In Brazil, UGT is typically treated with a six-month regimen, comprising rifampicin, isoniazid, pyrazinamide, and ethambutol for the first two months, followed by only isoniazid and rifampicin for the remaining four months. Extended treatment may be warranted in cases of HIV coinfection or kidney abscesses^11^. Earlier studies suggested extending treatment to 24 months for selected cases, particularly those with multidrug resistance^12^. Despite receiving appropriate antituberculosis therapy, the described case highlights the persistence of urological sequelae. This raises the question of whether prolonged treatment for UGT might help reduce or eliminate such complications, necessitating further investigation.

The main predisposing factors for genitourinary tract complications include non-specific symptoms, prolonged infections, lack of clinical suspicion of TB, and increased drug resistance^3^. Delay in diagnosing UGT may also stem from medical factors (inability to recognize the disease and lack of awareness of the low sensitivity of diagnostic tests)^13^. The most frequently reported complications include kidney damage, urinary strictures, and infertility^14^. In this case, the patient continued to experience significant changes in urinary frequency and mild pelvic discomfort. Urological evaluation confirmed the irreversibility of these sequelae.

## CONCLUSIONS

This case underscores the diagnostic and therapeutic challenges of UGT, particularly in the presence of non-specific symptoms and prolonged disease latency. Despite timely initiation of standard first-line anti-TB drugs and retreatment, the patient experienced irreversible urological sequelae, such as infundibular stenosis, polyuria, and nocturia, significantly affecting quality of life and daily functioning.

The findings highlight the importance of early suspicion, accurate diagnosis, and prompt intervention to minimize complications. Imaging modalities play a pivotal role in identifying infection sites and guiding treatment plans, and the persistence of sequelae raises critical questions about the adequacy of current treatment durations for UGT. Future studies are needed to evaluate the potential benefits of extended therapy in preventing long-term complications and improving patient outcomes in complex cases like this.
